# Expresso: A database and web server for exploring the interaction of transcription factors and their target genes in *Arabidopsis thaliana* using ChIP-Seq peak data

**DOI:** 10.12688/f1000research.10041.1

**Published:** 2017-03-28

**Authors:** Delasa Aghamirzaie, Karthik Raja Velmurugan, Shuchi Wu, Doaa Altarawy, Lenwood S. Heath, Ruth Grene

**Affiliations:** 1 Genetics, Bioinformatics, and Computational Biology (GBCB), Virginia Tech, Blacksburg, VA, 24061, USA; 2 Center for Bioinformatics and Genetics and the Primary Care Research Network, Edward Via College of Osteopathic Medicine, Blacksburg, VA, 24060, USA; 3 Department of Horticulture, Virginia Tech, Blacksburg, VA, 24061, USA; 4 Department of Computer Science, Virginia Tech, Blacksburg, VA, 24061, USA; 5 Department of Plant Pathology, Physiology, and Weed Science, Virginia Tech, Blacksburg, VA, 24061, USA

**Keywords:** ChIP-Seq, transcription factor, gene regulation, transcriptional regulation

## Abstract

**Motivation:** The increasing availability of chromatin immunoprecipitation sequencing (ChIP-Seq) data enables us to learn more about the action of transcription factors in the regulation of gene expression. Even though *in vivo* transcriptional regulation often involves the concerted action of more than one transcription factor, the format of each individual ChIP-Seq dataset usually represents the action of a single transcription factor. Therefore, a relational database in which available ChIP-Seq datasets are curated is essential.

**Results:** We present Expresso (database and webserver) as a tool for the collection and integration of available *Arabidopsis* ChIP-Seq peak data, which in turn can be linked to a user’s gene expression data. Known target genes of transcription factors were identified by motif analysis of publicly available GEO ChIP-Seq data sets. Expresso currently provides three services: 1) Identification of target genes of a given transcription factor; 2) Identification of transcription factors that regulate a gene of interest; 3) Computation of correlation between the gene expression of transcription factors and their target genes.

**Availability** **:** Expresso is freely available at http://bioinformatics.cs.vt.edu/expresso/

## Introduction

Chromatin immunoprecipitation (ChIP) is a method to investigate DNA-binding sites of DNA-binding proteins, such as transcription factors (TFs) ( Valouev *et al.*, 2008). ChIP can provide genome-wide information of *in vivo* protein-DNA interactions ( Kaufmann *et al.*, 2010). Therefore, it has become an important tool to assay TF-associated gene regulations ( Kaufmann *et al.*, 2010; Park, 2009; Valouev *et al.*, 2008). In a typical ChIP experiment, first the DNA-binding protein of interest is cross-linked to its binding sites. Then the chromatin is sheared, randomly, into short fragments and the protein-DNA complexes are purified by immunoprecipitation using a specific antibody against the DNA-binding protein of interest. Finally, genome-wide profiling of protein binding sites is produced by either genome-tiling arrays (ChIP-ChIP) or next-generation sequencing technologies (ChIP-Seq) ( Kaufmann *et al.*, 2010; Valouev *et al.*, 2008). Compared to ChIP-ChIP, ChIP-Seq provides high-resolution data with a better signal-noise ratio. ChIP-seq also requires less initial material and is more cost-effective ( Ho *et al.*, 2011; Kaufmann *et al.*, 2010; Valouev *et al.*, 2008). Therefore, ChIP-Seq has displaced ChIP-ChIP rapidly and is currently the most widely used technology for studying the action of transcription factors ( Park, 2009; Valouev *et al.*, 2008).

In contrast to the biomedical field, the use of ChIP-Seq in plant biology is limited ( Kaufmann *et al.*, 2010). For example, the GEO database ( https://www.ncbi.nlm.nih.gov/gds) currently contains 8,486 ChIP-Seq human datasets (as of October 2016), but has only 200 *Arabidopsis* datasets. The delay in the use of ChIP-Seq technology in plant research may be due to the specific properties of plant tissue, such as the presence of the cell wall and abundant secondary metabolites that affect the quality of protein-DNA complex extraction ( Kaufmann *et al.*, 2010). However, with the improvement of ChIP-Seq protocols and reduction of next-generation sequencing costs, an increasing number of plant scientists are choosing ChIP-Seq to study function of transcription factors in detail.

ChIP datasets currently available for *Arabidopsis* are isolated, fragmentary and they lack a uniform format. Thus a major gap exists between the capabilities of *in vitro* methods, such as ChIP Seq and the goal of understanding the complexities of transcriptional regulation. We report on the curation of the Expresso database to collect and integrate *Arabidopsis* ChIP-Seq data (available as peaks), which in turn can be linked to a user-provided *Arabidopsis* gene expression data. Expresso compiles 20 groups of selected *Arabidopsis* ChIP-Seq peak datasets downloaded from NCBI GEO or supplemental data of the corresponding paper. All collected ChIP-Seq peak datasets were re-analyzed by the Expresso processing pipeline to create a coherent and unified results which bridge the gap among multiple ChIP-Seq studies, and to provide a consensus access to TFs, target genes and DNA-binding motifs. In summary, instead of going though separate ChIP-Seq datasets, Expresso provides a more rapid and integrated method for the systematic study of the action of plant transcription factors.

## Methods

The Expresso computational analysis pipeline comprises preprocessing of peak loci reported by at each reference dataset, finding conserved motifs using MEME-suite ( Bailey *et al.*, 2009), identifying potential target genes for each transcription factor, and finally storing target genes and motifs linked to TFs into the database. Data-formatting primarily involves the extraction of a peak locus peak, peak summit and DNA sequences in fasta format from the *Arabidopsis thaliana* genome. Of the 50 datasets, almost all were found to be in distinct formats and only 20 had the peak information available either on GEO or at their supplemental material section of their corresponding published manuscript. We restructured the downloaded data into a unique format by extracting a specific set of information including: peak ID, chromosome number, peak start and end positions and genes in 1kbp distance of the peak summit. All the codes for preprocessing of the input data are available at Expresso GitHub page under “preprocessing”.

**Candidate target gene finding using motif search:** Given the chromosome number and peak start and end positions, the corresponding genomic sequence was extracted and trimmed, and then were subject to motif search using MEME-suite tool ( http://meme-suite.org/), with following parameters: -nmotifs 20 -minw 5 -maxw 30 -dna. While the distribution of the length of the untrimmed peak sequences of each dataset varied widely, the reported peak summit lengths were usually 200 to 500 bases long upstream and downstream from the middle of the summit ( Bailey *et al.*, 2009; Immink *et al.*, 2012; Valouev *et al.*, 2008). For a few datasets, the summit length was not provided in the article, so the largest summit length found, 500 bases, was used. Motif width was set to the length of the reported motif (if any). Otherwise, motif width was set to 5 to 30 bps, and significant motifs (E-value < 0.05) together with the candidate target genes possessing those motifs were uploaded to the database. Hence, a gene should have the following properties to be eligible to be uploaded to the database: i) should be among the target genes provided by a ChIP-Seq experiment, or within 1kbps distance of the peak summit ii) should have a significantly enriched motif in its peak binding site. Moreover, the presence of the motif found by MEME was validated by the reported motif in the reference paper. If the reported motif was not found using the MEME search tool on the peak sequences, the resulting motifs were not uploaded to the database.

## Results

Expresso provides a user-friendly environment to facilitate exploring different transcription factors and target genes through motif analysis. ChIP-Seq experiments in Expresso are available under the “Experiments” tab. Expresso currently provides three services for identifying: 1) the target genes of a given transcription factor, 2) the transcription factors that regulate genes of interest and 3) the correlation of gene expression between transcription factors and their target genes.

**Identifying candidate target genes for a transcription factor (see “Transcription Factors” on the Expresso website:** http://bioinformatics.cs.vt.edu/expresso/?q=node/3): Users can select a transcription factor from the list of available transcription factors to view potential target genes. Since target genes for each transcription factor have been compiled from the peaks and motifs data, users can change the cut-off for the motif E-value. The default E-value is set to 0.05. A short functional description (along with a link to TAIR10) and the GEO id for the reference ChIP-Seq experiment is provided for each potential target gene. For example, searching for target genes of TOC1 transcription factor results in 298 genes that have at least one significantly enriched motifs at least one peak located close to their transcription start site.

**Identifying potential transcription factors regulating a target gene (see “Genes” on the Expresso website:** http://bioinformatics.cs.vt.edu/expresso/?q=node/4): Users can enter a gene or multiple genes and Expresso finds all the transcription factors that might regulate that gene together with the binding motif for that TF. For example, SGP2 (AT3G21700) gene is potentially transcriptionally regulated by PIF3 and KAN1.

**Exploring gene expression data:** Users can upload gene expression data and Expresso finds genes and transcription factor pairs present in Expresso database and performs Pearson correlation analysis on their corresponding expression data. Upon submission of the gene expression, a task id is assigned to this job. Users need to keep the task id to retrieve the results or check the status of their job. If they provide an email address, they will be notified when the results get ready. To demonstrate the application of correlation analysis on finding potential TF-target gene pairs, a RNA-Seq dataset ( Segaran, 2007) has been added to Expresso as a demo (see “Gene Expression” on the Expresso website: http://bioinformatics.cs.vt.edu/expresso/?q=node/5). 100 genes (including some transcription factors) were selected randomly from this dataset, which has expression values for genes from different *Arabidopsis* tissues: leafs, seeds, roots and flowers. 54 genes were found to be target genes of transcription factors in Expresso. 33% of the uploaded genes were found to be targets genes of multiple transcription factors. The correlation of gene expression between a transcription factor and its target genes can be used for inferring their relationship. For example, three out of four target genes of PIF3 show high correlation with the expression of PIF3, although one gene was found to have a negative correlation (R=-0.92). The fact that their expression patterns are correlated with PIF3, suggests that PIF3 plays a dominant role in regulating these three target genes. However, AT3G21700 was found to have a low correlation with PIF3, which suggests that there might be other transcription factors that challenge PIF3 in the regulation of AT3G21700.

## Conclusions

ChIP-Seq is a powerful technology that aides in the study of the action of transcription factors, predicting a given transcription factor's target genes and corresponding conserved binding motifs ( Ho *et al.*, 2011; Kaufmann *et al.*, 2010; Park, 2009; Valouev *et al.*, 2008). The Expresso database is curated to integrate several available ChIP-Seq datasets. Expresso provides an easy access to 1) potential targets of a given transcription factor and their possible binding sites; 2) candidate transcription factors regulating several genes of interest; 3) correlation analysis of TF and target gene pair using the user’s input gene expression data. Taken together, Expresso facilitates an easy access to several ChIP-Seq experiments, making the study of the transcriptional regulation in the cells easier in the context of interaction among several transcription factors.

## Software and data availability

Expresso is freely available online: http://bioinformatics.cs.vt.edu/expresso/

Source code available at: https://github.com/doaa-altarawy/Expresso/tree/2.0.0

Archived source code as at time of publication: doi, 10.5281/zenodo.399501 ( Altarawy, 2017).

License: MIT

All datasets were publicly available and were downloaded from GEO DataSets. The list of ChIP-Seq datasets available in Expresso is available at ‘Experiments’ section on Expresso. The list of transcription factors and target genes can be downloaded in the text format.
