# Supplementary material for: Expresso: A database and web server for exploring the interaction of transcription factors and their target genes in Arabidopsis thaliana using ChIP-Seq peak data
Source: F1000Res. 2017 Mar 28;6:372. [Version 1] doi: 10.12688/f1000research.10041.1 (PMC5414811; doi:10.12688/f1000research.10041.1)
Supplement: Peer review history

# Peer review: Referee response for version 1

**DOI:** 10.5256/f1000research.10818.r22402

**Published:** 2017-05-02

**Authors:** Sakiko Okumoto

**Affiliations:** 1 Department of Soil and Crop Sciences, Texas A&M University, College Station, TX, USA

In this manuscript, the authors created a web-based interface which hosts ChIP-seq data from 20 published experiments, and allows the users to 1) access the compiled lists of TF targets that met the criteria set by the authors, 2) identify the transcription factor(s) that regulate his/her gene of interest, and 3) perform co-expression analyses with user-provided RNAseq data.

 Although there are other web-based services that allow at least part of what is described above, the authors argue that this is the first platform that provides a uniform format for 20 genes. I would like to agree with the authors about the value of such a format.

 Of the above three functions, the first two are fairly straightforward and seems to function as expected. However, I feel that the description found in the manuscript about the co-expression analysis did not contain enough information.

 The authors provide a list of 100 genes with their FPKM values in 4 different tissues as a demo. The manuscript describes the set as “100 genes (including some transcription factors) were selected randomly from this dataset”. When comparing this list of 100 with the list of TFs in this database however, I see that 16 out of 20 TFs in the database are included in the list of 100 genes. This seems more than “some” to me- please describe specifically. When I run the demo, none of the 4 that are not in the list of 100 are found to be co-expressed with any of the genes. I think that would make sense because I don’t see how one can deduce co-expression between a given gene and a TF it the TF is not expressed in the data set. If this is indeed a requirement, I would like to see that stated in the manuscript.

 Also, typically how many tissues/times would need to be in the dataset? (When I remove one of the columns in the demo set I don’t get any hits, probably due to less statistical power of the data set.) It would be beneficial for the readers to know the approximate number experiments needed for a correlation analysis.

 In general, I would really appreciate if the authors could explain how co-expression analysis works – does it first perform co-expression analyses within the genes in the uploaded dataset, identify the TFs in the data base, then select the ones that have the consensus motif? A lay-friendly flow chart would be much appreciated.

 Also, it would be nice if the algorithm identified the motif and the distance from the ATG in the co-expressed genes.

 Minor points include:

- On the “Experiment” tab- for each TF, would you please include the link to the original publication? One can trace back using GEO NCBI, but it would be easier for the users if the publication is included as an additional column.
- To demonstrate the application of correlation analysis on finding potential TF-target gene pairs, a RNA-Seq dataset (Segaran, 2007) has been added to Expresso as a demo” I am fairly certain that the reference provided here is wrong.
- Methods “a peak locus peak” a peak locus?

I have read this submission. I believe that I have an appropriate level of expertise to confirm that it is of an acceptable scientific standard.

# Peer review: Referee response for version 1

**DOI:** 10.5256/f1000research.10818.r21320

**Published:** 2017-04-18

**Authors:** Asa Ben-Hur

**Affiliations:** 1 Department of Computer Science, Colorado State University, Fort Collins, CO, USA

The authors have created a useful resource that provides unified access to a large number of ChIP-seq experiments in arabidopsis. The database has useful functionality that would be useful for exploring TF binding. Each function of the database has example data that allows users to try it out easily, and the pipeline is available through github.

 The following should be addressed:

1. The major issue with the paper is that there is an existing similar resource called ChIPBase (see citations below ^1^^,^^2^). The authors should cite it and compare their database with it, as it's not obvious what Expresso is adding to what it provides.
2. A figure that summarizes your data analysis pipeline would be beneficial (I saw such a figure on the Expresso website).
3. In the section on motif finding: did you focus on promoter regions for the peaks, and if so, how were those defined? The motifs generated by MEME were compared against those in the corresponding papers, and no motif was added if MEME did not detect a motif. How often did that happen? MEME occasionally misses motifs, and other tools could have possibly found those motifs.
4. In the expression section, please suggest how the user should measure expression to provide good results. For that matter, please provide information on how expression of the TFs is quantified.

Minor comments:

 There are some grammar issues that need to be fixed - see below.

1. In the introduction you write that "ChIP can provide genome-wide information...". That is true when performed as ChIP-ChIP or ChIP-seq.
2. TF-associated gene regulations --> regulation
3. "ChIP-Seq in plant biology is limited": I think you meant that it hasn't been as widely used as in mammalian systems.
4. I did not buy your explanation of the delay in adoption of ChIP-seq in plant research. Plant research tends to be a few steps behind, and furthermore, many more people study human than arabidopsis.
5. "All the codes for preprocessing" --> all the code for preprocessing
6. "results in 298 genes that have at least one significantly enriched motifs at least one peak located close to their transcription start site." something unclear here - "one significantly enriched motifs at least one peak" - should there be an "or" or "and" enriched motif AND at least one peak?  And the word motif should be singular, and refer to a motif hit/occurrence.
7. targets genes --> target genes
8. "can be downloaded in the text format" --> in text format

I have read this submission. I believe that I have an appropriate level of expertise to confirm that it is of an acceptable scientific standard.

## References

[1] ZhouKRLiuSSunWJZhengLLZhouHYangJHQuLH: ChIPBase v2.0: decoding transcriptional regulatory networks of non-coding RNAs and protein-coding genes from ChIP-seq data. *Nucleic Acids Res*.2017;45(D1) : 10.1093/nar/gkw965 D43-D50 10.1093/nar/gkw965 27924033PMC5210649

[2] YangJHLiJHJiangSZhouHQuLH: ChIPBase: a database for decoding the transcriptional regulation of long non-coding RNA and microRNA genes from ChIP-Seq data. *Nucleic Acids Res*.2013;41(Database issue) : 10.1093/nar/gks1060 D177-87 10.1093/nar/gks1060 23161675PMC3531181

# Peer review: Referee response for version 1

**DOI:** 10.5256/f1000research.10818.r21317

**Published:** 2017-04-18

**Authors:** Nicholas J. Provart

**Affiliations:** 1 Department of Cell and Systems Biology, University of Toronto, Toronto, ON., Canada; 2 Centre for the Analysis of Genome Evolution and Function, University of Toronto, Toronto, ON., Canada

In principle the Expresso database will be useful to plant researchers. I would like to see a couple of things: what about mention of other databases like AGRIS at OSU and Cistome/ePlant at the BAR? Do these capture the kinds of interactions the authors are describing? Another is HRGRN (http://plantgrn.noble.org/hrgrn/). What are the disadvantages/limitations of these vis a vis Expresso?

 Another thing that would a “nice-to-have” would be to include the Ecker Lab’s recent, extensive DAP-seq data set, which the authors ( https://www.ncbi.nlm.nih.gov/pubmed/27203113) show to be quite concordant with existing Chip-Seq data. These data are more extensive than the fairly limited number of Chip-seq data sets that Aghamirzaie et al. have collated.

 I tried out the software, which worked as promised. The functionality was somewhat basic. It would be quite easy to use table.js or similar on the “Genes” search output page, or on the “Gene Expression” output page to be able to sort the table of favourite genes with their targets as a user expects to be able to do, or to sort by Pearson correlation. It might be nice to let users know how to download the “Genes” search results and load them into Cytoscape in a tutorial section. I was unable to download a file of TFs binding to my favourite genes (URL http://bioinformatics.cs.vt.edu/expresso/Expresso_Codes/getResFile_Genes.php) – the page returned an error of “Unable to select database”.

 “Run Demo” did not work on the “Gene Expression” page, or at least I thought it didn’t until I realized I had to scroll down to see the results, which appeared...but off the bottom of my screen…a little Javascript autoscroll to that section would be helpful after the calculation has finished.

Typos/grammar

 In general: it’s ChIP-chip, not ChIP-ChIP (the first ChIP is for Chromatin Immuno-Precipitation, the second “chip” refers to microarray)

 Be consistent: either ChIP-seq or ChIP-Seq (we see ChIP-seq, ChIP-Seq, and ChIP Seq in the paper)

 Candidate target gene finding section: “the corresponding genomic sequences were extracted and trimmed” (“sequences” should be plural as multiple genomic sequences are analyzed, no?)

 Candidate target gene finding section: “Otherwise, motif width was set to be between 5 to 30 bp” (“…to 5 to 30” is awkward)

 Bottom of page 3: (“along with a link to TAIR10”) – TAIR10 refers to the 10th genome build. I’d say rather “along with a link to TAIR”. It might be nice to add a link to the Araport record for a given gene too.

 Top of page 4: “…motifs in at least one peak located close to…” (missing “in”?)

 Midway down page 4: “…the results are complete.” (instead of “get ready”)

I have read this submission. I believe that I have an appropriate level of expertise to confirm that it is of an acceptable scientific standard, however I have significant reservations, as outlined above.
